# Plasmonic Nanoparticles as Optical Sensing Probes for the Detection of Alzheimer’s Disease

**DOI:** 10.3390/s21062067

**Published:** 2021-03-16

**Authors:** María Paz Oyarzún, Andreas Tapia-Arellano, Pablo Cabrera, Pedro Jara-Guajardo, Marcelo J. Kogan

**Affiliations:** 1 Departamento de Química Farmacológica y Toxicológica, Facultad de Ciencias Químicas y Farmacéuticas, Universidad de Chile, Dr. Carlos Lorca Tobar 964, Independencia, 8380000 Santiago, Chile; maria.oyarzunm@usach.cl (M.P.O.); atare_28@ciq.uchile.cl (A.T.-A.); pablo.cabrera@ug.uchile.cl (P.C.); pedro.jara@ug.uchile.cl (P.J.-G.); 2 Advanced Center for Chronic Diseases (ACCDIS), Sergio Livingstone #1007, Independencia, 8380492 Santiago, Chile

**Keywords:** nanoparticle based-plasmonic sensors, Alzheimer’s disease, AD biomarkers, kinetics of aggregation, amyloid-β peptide, tau protein, SERS detection, fluorescence detection, colorimetric detection, LSPR detection, in vivo imaging detection

## Abstract

Alzheimer’s disease (AD), considered a common type of dementia, is mainly characterized by a progressive loss of memory and cognitive functions. Although its cause is multifactorial, it has been associated with the accumulation of toxic aggregates of the amyloid-β peptide (Aβ) and neurofibrillary tangles (NFTs) of tau protein. At present, the development of highly sensitive, high cost-effective, and non-invasive diagnostic tools for AD remains a challenge. In the last decades, nanomaterials have emerged as an interesting and useful tool in nanomedicine for diagnostics and therapy. In particular, plasmonic nanoparticles are well-known to display unique optical properties derived from their localized surface plasmon resonance (LSPR), allowing their use as transducers in various sensing configurations and enhancing detection sensitivity. Herein, this review focuses on current advances in in vitro sensing techniques such as Surface-enhanced Raman scattering (SERS), Surface-enhanced fluorescence (SEF), colorimetric, and LSPR using plasmonic nanoparticles for improving the sensitivity in the detection of main biomarkers related to AD in body fluids. Additionally, we refer to the use of plasmonic nanoparticles for in vivo imaging studies in AD.

## 1. Introduction

Alzheimer’s disease (AD) is a neurodegenerative disease (NDD) and is the most common kind of dementia, with a typical clinical presentation characterized by loss of memory and cognitive impairment. AD involves different processes that finally lead to both synaptic and neuronal loss in brain regions such as the cerebral cortex and subcortical area, triggering cognitive impairment, memory loss, brain dysfunction, and behavioral disorders [1,2].

The accumulation and aggregation of the amyloid-β peptide (Aβ) as extracellular deposits and the formation of intraneuronal aggregates of hyperphosphorylated tau protein (P-tau) as neurofibrillary tangles (NFTs) in the brain, associated by inflammation and oxidative stress processes [3] have been described as the pathological hallmarks of AD. In addition, more recently several studies have suggested that soluble Aβ oligomers (AβOs) are the most toxic species formed, leading to a synaptic dysfunction [4,5,6,7].

Since these Aβ species have been identified according to their direct participation in the development of AD, various biomarkers could be useful to detect AD in the predementia phase [8] and to start an effective treatment to prevent further deleterious damage on the brain and neuronal loss. The core cerebrospinal fluid (CSF) biomarkers: total tau (T-tau), phosphorylated tau (P-tau) and the 42-amino acid form of Aβ (Aβ_1–42_) are the most frequently used as AD-related biomarkers [9,10]. Thus, low concentration levels of Aβ_1–42_ can be detected in CSF at the onset of the disease, whereas concentrations of T-tau and P-tau are increased and are detected in long-term courses [8,11]. On the other hand, an increased Aβ_1–42_/Aβ_1–40_ ratio in CSF indicates increased levels of Aβ_1–42_ [12], which is more prone to aggregate than Aβ_1–40_. Aβ_1–42_ has been related to increased neurotoxicity of Aβ and is considered a reliable index due to its high diagnostic accuracy [10].

Moreover, some studies have focused on genetic components that can be considered as risk factors. In fact, the presence of high levels of apolipoprotein E4 allele (ApoE4) has been considered as a major genetic risk factor of AD, whereas high levels of apolipoprotein E2 allele (ApoE2) would exert a protective role [13,14].

In this context, the development of analytical devices for rapid, sensitive, reliable, and low-cost methods for the simultaneous detection of various AD biomarkers in primary stages of the disease could contribute to improve understanding of the molecular mechanisms underlying the pathophysiology of this disease [3].

In particular, the fabrication of sensors for the detection of biological analytes with high sensitivity and selectivity has significantly increased in the last decade. In this field, nanomaterials have become an interesting and useful tool due to their versatile uses, such as the ability to immobilize biorecognition molecules on their surface, the high surface-area-to-volume ratio, or the capability to amplify signals, which could facilitate recognizing and sensing molecules of biological interest [3,15]. Among these nanomaterials, “plasmonic metal nanoparticles” (including gold, silver, platinum, etc.) are those that support a localized surface plasmon resonance (LSPR) phenomena, allowing them to absorb and scatter the light in different ways. These interesting and useful properties making them excellent candidates for broad applications in the nanomedicine field, as diagnostics (sensing), imaging, or therapeutics (therapy agents) [16].

**Figure 1:**
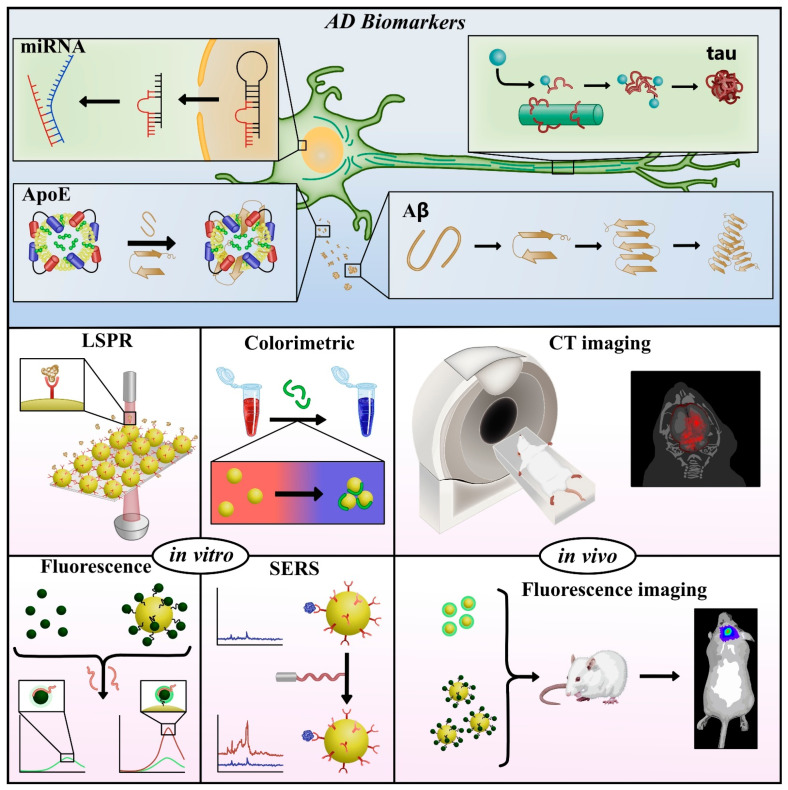
General scheme of optical detection platforms that use plasmonic nanoparticles for detecting in vitro Alzheimer’s disease (AD) core (Aβ and tau protein) and other biomarkers associated with the development of AD. Due to AD biomarkers are at trace levels in biological fluids such as blood and cerebrospinal fluid (CSF), the development of sensitive and specific methods is highly necessary to detect these biomarkers and establish an early diagnosis and progression of this disease. Considering these aspects, different optical techniques as SERS, fluorescence, colorimetric, and LSPR have been proposed for the detection of AD biomarkers. Additionally, molecular in vivo imaging techniques, such as magnetic resonance imaging (MRI) and X-ray computed tomography (CT), have been used to observe structural changes in the brain with AD. Thus, the synergy between the detection of AD biomarkers in biological fluids and imaging techniques could provide an early diagnosis of AD.

The present review focuses on the advances made during the last decade (2010–2020) in the development and application of plasmonic nanoparticles in optical sensors for in vitro detection of main AD biomarkers in artificial and real samples. In addition, we refer to some studies of the application of these nanoparticles for imaging in in vivo diagnosis, as illustrated in Figure 1. Finally, this review brings into focus future perspectives of the biosensing of AD.

## 2. Relevant AD Biomarkers

Biomarkers constitute physiological, biochemical, and anatomical variables that can be measured in vitro and in vivo and indicate specific features of disease-related pathological changes [17]. Several biomarkers have been reported to have a specific role in the development of AD, such as Aβ peptide in cerebral amyloid deposition and tau protein in neurodegeneration.

In this section, we summarize the characteristics of core AD biomarkers (Aβ and tau) involved in the formation of senile plaques and NFTs, respectively, as well other AD biomarkers are mentioned, such as ApoE, miRNA, etc. 

### 2.1. Amyloid-β Peptide (Aβ)

**Figure 2:**
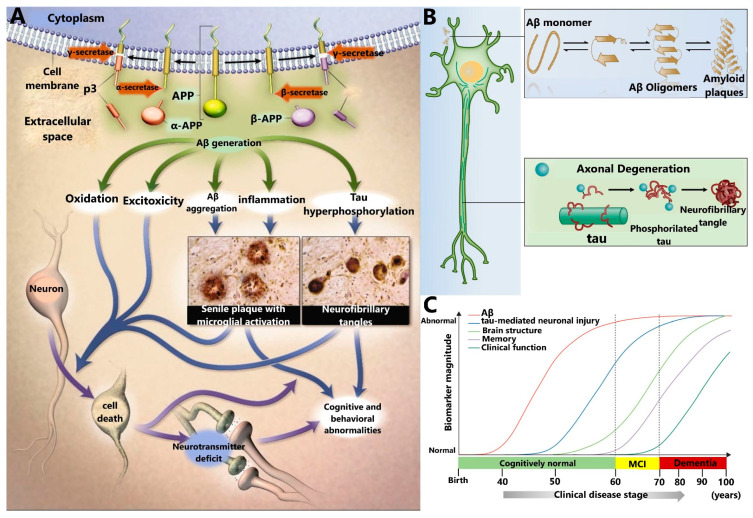
The putative amyloid cascade hypothesis and neuropathological hallmarks of AD. (**A**) This hypothesis states that AD progresses from the generation of the Aβ peptide from the amyloid precursor protein (APP), through multiple secondary steps, until reaching cell death. From ref. [39] Copyright © 2021 Massachusetts Medical Society. Reprinted with permission from Massachusetts Medical Society. (**B**) In the amyloid cascade hypothesis, Aβ monomers exist in equilibrium with aggregates of higher molecular mass (dimers, trimers, and oligomers), which finally form a deposit of amyloid plaques at the extracellular level. On the other hand, detachment of P-tau from axonal microtubules would destabilize them, resulting in axonal degeneration at the intracellular level and forming the neurofibrillary tangles (NFTs) at the intracellular level. (**C**) Hypothetical model showing the time-dependent changes in the levels of AD biomarkers. Levels of Aβ and tau in CSF are identified by PET (positron-emission tomography) and fluorodeoxyglucose-PET. Brain structure is visualized using structural magnetic resonance imaging. MCI: mild cognitive impairment. Republished with permission of Royal Society of Chemistry from ref. [40].

Among the biomarkers that are currently known for AD diagnosis, the most relevant one is the Aβ peptide, showing a key participation in the development of AD, as described in the amyloid cascade hypothesis [4,18,19] shown in Figure 2A. This hypothesis posits that Aβ is produced by the processing of the amyloid precursor protein (APP), a transmembrane protein located in the neuronal cell membrane [20,21]. Under physiological conditions, Aβ is cleared from the brain through the CSF; however, under pathological conditions, Aβ suffers a state of aggregation through the formation of different intermediate species (Figure 2B), such as dimers, trimers, oligomers (AβOs), protofibrils (AβPFs), and fibers (AβFs), which eventually are deposited as senile plaques [22,23]. The latter has been observed in the brain of AD patients through imaging techniques used in clinical diagnosis.

AD has been hypothesized to begin 15–20 years (preclinical stage) before the first clinical symptoms are manifested [11]. In the primary stage, Aβ accumulation and aggregation promote inflammatory processes and aggregation of other proteins, such as the tau protein, increasing neurodegenerative damage [19,24]. At this stage, the patient is cognitively normal and does not reflect any symptom maintaining this condition for decades. Later, Aβ peptide aggregates into senile plaques at the extracellular level while tau protein aggregates into neurofibrillary tangles at the intracellular level, provoking several symptoms at the brain level that are altogether termed mild cognitive impairment. Thus, both kinds of aggregates start to trigger neuronal death and brain injuries modifying the structure of the brain causing the first symptoms of the disease. When these symptoms get worse, progress to dementia, which is the final stage of AD (Figure 2C).

Therefore, the early detection of these species as clinical biomarkers of AD is very attractive and important to provide timely treatment and limit disease progression.

In particular, the two common isoforms of Aβ, soluble Aβ_1–40_ and insoluble Aβ_1–42_, are thought to be critical elements in AD pathogenesis. The concentration of soluble AβOs is increased in CSF of AD, compared to control subjects [25] and since this aggregate has been described as highly toxic, it may serve as a biomarker for AD diagnosis [25].

As senile plaques are composed mainly of Aβ_1–42_, it has been observed that a decrease in the concentration of free Aβ_1–42_ in CSF appears to be the earliest detectable biomarker attributed to the development of AD (before clinical symptoms); therefore, its detection could be an interesting strategy for an early diagnosis of this disease. On the other hand, the concentration of Aβ_1–40_ has been reported to be unaltered in AD; however, the concentration of Aβ_1–42_ with respect to Aβ_1–40_ (Aβ_1–42_/_1–40_ ratio) in CSF is higher than the concentration of Aβ_1–42_ alone in patients with AD [26]. In recent years, blood analysis has received enormous attention as a non-invasive method for the detection of AD biomarkers, however, it exhibits some limitations, such as the complexity of its matrix, the low amount of proteins from the brain that reach the bloodstream, and the high probability that these proteins become rapidly degraded, metabolized or eliminated from the body [27]. In addition, the majority of these developed methods have failed to validate the clinical utility of quantifying AD biomarkers in blood. 

### 2.2. Tau Protein

Tau is a key protein for microtubule stability in neurons and plays an important role within cell structure and neuronal traffic. Tau protein dysfunction in the brain has been associated with NDDs as AD and other tauopathies. Abnormally, P-tau mainly contributes to forming NFTs inside the brain, which are insoluble aggregates that generate paired helical and straight filaments [8], as shown in Figure 2B.

NFTs, in conjunction with extracellular Aβ aggregation, have been described as two valuable biomarkers for AD diagnosis [28]. 

In addition, high concentrations of T-tau and P-tau in CSF-due to cortical neuronal loss and cortical tangle formation, respectively- and low concentrations of Aβ_1–42_ due to cortical amyloid deposition, have been described as useful biomarkers for AD diagnosis [29,30]. However, due to the CSF extraction is an invasive procedure for patients, the detection of tau levels in blood has also been proposed. More recent research has demonstrated that blood concentration levels of P-tau at threonine 181 (P-tau_181_) could help diagnosis and differentiation of AD from other NDDs [31].

For T-tau and P-tau proteins, the concentration levels in biological fluids that have been reported are very low (195 pg mL^−1^ or 4.3 pM in CSF [29]). For this reason, the development of highly specific and sensitive analytical techniques is essential. However, currently, the fabrication of new sensing platforms for tau detection has been relatively less than Aβ peptide detection platforms.

### 2.3. Other AD Biomarkers

The biomarkers aforementioned are some of the most studied and known for AD due to their participation in the development of this disease; however, a unique biomarker to diagnose AD with high accuracy has not yet been discovered. In this context, the detection of multiple biomarkers has been reported [32,33].

Whereas the function of Aβ and tau in AD have been described in the literature, the apolipoprotein E4 (ApoE4) is indicated as the most prevalent genetic risk factor in AD. ApoE4 can promote the accumulation of Aβ in the brain, stimulating its permanence and, subsequently, its aggregation. In fact, high ApoE4 levels in the brain have been associated with a high-risk genetic factor, whereas ApoE2 and ApoE3 have mainly been associated with a protective factor [13,34]. Also, gene mutations have been associated that cause autosomal dominant familial AD are APP on chromosome 21, presenilin-1 (PSEN1) on chromosome 14, and presenilin-2 (PSEN2) on chromosome 1 [35]. On the other hand, some specific miRNAs including miR-9, miR-137, miR-181, and miR-29, associated with immune response and inflammation, have also been related to AD [36].

Due to the large number of novel AD biomarkers that have been reported and reviewed in the literature [32,37,38], in the next sections we will focus on optical detection methods reported using metal nanoparticles for sensing Aβ, tau, and other biomarkers in clinically relevant fluids.

## 3. Optical Properties of Plasmonic Metal Nanoparticles

**Figure 3:**
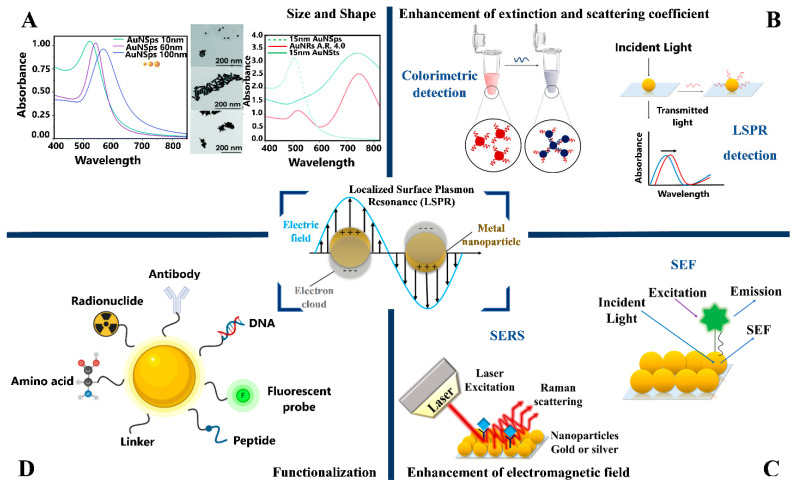
Localized surface plasmon resonance (LSPR) in plasmonic metal nanoparticles. (**A**) UV-Vis absorption spectra of gold nanospheres (AuNSps) showing the effect of size in LSPR and gold nanoparticles with various geometries: spheres (AuNSps), rods (AuNRs), and stars (AuNSts). Modified and republished with permission of Royal Society of Chemistry from ref. [63]. (**B**,**C**) The LSPR on metal NPs generates different effects, so they can be applied in different optical detection techniques, such as colorimetric, LSPR, SERS, and SEF. (**D**) The surface of plasmonic NPs can be modified with a wide variety of molecules and biomolecules.

The optical properties of metal nanoparticles (NPs) are very important for many of their applications. Noble NPs with a negative real and small positive imaginary dielectric constant, support a localized surface plasmon resonance (LSPR), generated by a collective oscillation of surface conduction electrons that strongly couple with electromagnetic radiation at specific wavelengths (Figure 3, central image) [41,42,43]. From the interaction of the NPs with incident light, a part of these photons are absorbed and others scattered in different directions [42,44]. Hence, one or more UV-vis extinction bands in the electromagnetic spectrum observed in NPs suspensions include both absorption and scattering components, which are highly enhanced when LSPR is excited [42,45].

The optical cross-sections (absorption and scattering) in spherical NPs has been calculated through Mie’s theory that describes the scattering of electromagnetic wave for homogeneous spherical particles solving Maxwell’s equations [46]. Furthermore, modifying Mie’s theory, it has been possible to include spheroids [47] and for other geometries, a discrete dipole approximation (DDA) has been applied [45].

As a result, the LSPR of NPs is very sensitive to changes in chemical composition, size, shape, or functionalization as well as dielectric permittivity of the environment where they are dispersed [42,43,48]. The chemical composition directly affects the interaction of the NPs with the light, so only noble metals such as Au, Ag, Cu, etc. have an LSPR band in the UV ultraviolet (UV)-visible and near-infrared region (NIR) [49].

The size and shape of NPs are important parameters that allow one to control a wide spectral tunability of LSPR along the UV-vis and NIR of the electromagnetic spectrum [44]. For example, gold nanospheres (AuNSps) with an average size of 16 nm have an absorption band at 520 nm, while 60 nm is located at 535 nm. For silver nanospheres (AgNSps) with a diameter of 5 nm, the LSPR band is at 390 nm and with 60 nm at ~400 nm [42].

For non-spherical NPs, different plasmon modes are observed, being the anisotropic NPs (e.g., rods, plates, prisms, stars) useful for biomedical applications, due to their LSPR is observed in the NIR where the biological transparency window is located [49,50]. For example, in gold nanorods (AuNRs) two resonance peaks are observed, which correspond to a weak transverse band, located in the visible range and a strong longitudinal resonance, located in the NIR region. In irregular-shaped NPs as stars, cubes, and others is common to observe broadened plasmon resonance peaks over a wide range of the electromagnetic spectrum due to a more complicated interaction with the light [49,51]. An example of changing the LSPR in UV-vis-NIR spectra by varying the size and shape are shown in Figure 3A.

Due to the absorption and scattering coefficients of NPs colloids that support LSPR can be several orders of magnitude higher compared to common organic molecules (e.g., 27 × 10^8^ M^−1^ cm^−1^ for 13 nm AuNPs [52]) and displaying intense colors in the visible region, make them suitable for example in colorimetric sensors (Figure 3B) which through the visualization of the color change for the naked-eye of the aggregation of NPs induced by a specific analyte, allows its detection [53]. Consequently, the NPs-based systems can be characterized by optical spectroscopy based on extinction or scattering measurements [44].

The high scattering makes plasmonic NPs suitable for different scattering detection techniques, for example, optical microscopy-based in darkfield and backscattering mode which allows their application in bioimaging. In biosensing, the sensitivity of LSPR to changes in the local refractive index originates from so-called LSPR sensors (Figure 3B).

The amplification of the local electromagnetic field (with increments of even above 10^2^ times in some cases [42]), induced by excitation of LSPR in the NPs produces the so-called “hot-spots”, which occur in nanoparticle aggregates or regions close to the surface of NPs, such as at sharp edges and tips in single NPs [44]. These hot spots can increase the fluorescence or Raman signals of molecules or substances localized at a certain distance from the surface of the NPs, leading to field enhancement applications as surface-enhanced fluorescence (SEF) and surface-enhanced Raman scattering (SERS), respectively [44,49,53], (Figure 3C).

In addition to their interesting optical properties influenced by composition, size, shape, refractive index, the NPs have other advantages as chemical stability, easy functionalization, large surface area, and the high surface-to-volume ratio [15]; making them useful and versatile materials in nanomedicine area, where usually, for in vitro sensing, gold (AuNPs) and silver (AgNPs) of different sizes and shapes are the most used because they absorb and scatter light in the 350–900 nm spectral range. Moreover, for in vivo studies, AuNPs are the most applied due to their satisfactory biological compatibility [15,48]. Convenient functionalization of plasmonic NPs with a wide variety of functional groups as antibodies, ligands, drugs, DNA, peptides (Figure 3D) [54], has allowed the development of new nanosystems for sensing of biomarkers of different diseases [3,55,56], diagnostic imaging [57,58], targeted drug delivery [59,60,61] and therapy for the treatment of cancer or neurodegenerative diseases [62].

## 4. Plasmonic Biosensors for In Vitro Optical Detection of AD Biomarkers

Due to the need for an early diagnosis of AD, the development of new platforms for the detection of biomarkers in biological fluids that present sensitivities that apply to the clinic have been continuously sought, within which those based in biosensing technology have become relevant in recent years.

A biosensor is defined as an analytical device composed of a bioreceptor (e.g., antibodies, DNA, cells, fluorescent molecules, etc.), which can recognize a biological target and a transducer that converts a biological response from a biorecognition interaction or reaction into a measurable signal, which can be optical, electrical, magnetic, thermal, etc. [3,8,10]. To provide a sensitive and selective detection, the bioreceptor should exhibit a high affinity or dissociation constant (Kd) towards the specific analyte. It has been reported that bioreceptors with Kd ~10^−15^ M can recognize analytes down to fM concentration level [40]. In this context, the most well-established bioreceptors with high dissociation constants are antibodies (10^−8^–10^−12^ M) [64] and aptamers (10^−6^–10^−12^ M) [40]. Although antibodies are widely used as sensing platforms, some disadvantages have been described such as low stability and higher lot-to-lot variations; on the other hand, aptamers are a promising alternative due to their features as high stability at room temperature, low cost, ease of synthesis, among others [40]. Antibodies and aptamers are the bioreceptors commonly used for sensing AD biomarkers. Other less common alternatives that have emerged recently are fluorescent probes, peptides, and other small molecules, and also label-free platforms.

The incorporation of different types of nanomaterials in biosensors has been considered as a fundamental parameter for enhancing analytical performance characteristics (precision, accuracy, sensitivity, selectivity, etc.). Particularly, plasmonic NPs, have been integrated into many biosensing platforms mainly due to their optical properties already described in Section 3, since they promote an increase in detection sensitivity, which is observed in the lower limits of detection (LOD) of several orders of magnitude reached [3]. In addition, reliability and robustness are other analytical parameters to be considered for the development and potential clinical application of a sensor [65].

Optical biosensors are the most common sensors containing a bioreceptor element integrated with an optical transducer system. Thus, these optical biosensing platforms measure the interaction between the target analyte and the bioreceptor through changes in Raman signals, absorbance, fluorescence, LSPR, or color changes [40]. For example, the conventional enzyme-linked immunosorbent assay (ELISA) test is a type of optical sensor based on absorption and/or fluorescence, with LODs in the pM range, however, several of the plasmonic biosensors described in the following subsections have achieved sensitivities at fM level.

In the following subsections, we will describe the current advances reported in the development of optical biosensors for different AD biomarkers incorporating mainly plasmonic metal NPs (AuNPs, AgNPs) and hybrid systems, nanowire arrays, nanogap shells structures among others, with in vitro applications such as biological fluids (CSF, blood, etc.) using SERS, SEF, colorimetric sensing and surface plasmon resonance (LSPR) detection.

### 4.1. Surface-Enhanced Raman Scattering (SERS) Detection

Optical detection techniques based on SERS represent a powerful tool for biosensing applications. The large electromagnetic field generated by the presence of “hot-spots” amplifies the Raman signals of SERS probes binding to a target analyte under the excitation by electromagnetic radiation [66]. While it is generally expected that the strongest SERS enhancement when the frequency of incident photon coincides with the surface plasmon resonance band (LSPR) of the NPs (on-resonance field enhancement), it has also been observed on immobilized nanostructures that the maximum Raman signal intensity occurs if the plasmon band is red-shifted compared to the laser excitation wavelength [67]. Similar results have been observed for AuNRs with different aspect ratios [68], and gold nanotriangles (AuNTs) [69,70], where the correlation between size and SERS enhancement is studied, exhibiting the highest SERS activity under off-resonance conditions, explained by a competition between SERS enhancement and extinction [71].

The nominal performance indicators as LODs and the known enhancements factors (EFs) are commonly used to compare efficiency among different SERS sensing platforms.

For example, for non-aggregated colloidal dispersions, the so-called SERS analytical enhancement factor (AEF) presents reported values between 10^4^−10^5^. NPs supported on a surface or nanostructured substrates in a dry state, the reported substrate enhancement factor (SEF or EF) are in ranges from 10^5^ to 10^6^ [65].

For this reason, the use of different plasmonic functional nanostructures as individual nanocrystals (e.g., spherical, triangular, rod or star-shaped) with intrinsic hot spots; hollow/nested or core-shell morphologies with nanoscale interior gaps, branched and film-coupled NPs, disordered assemblies as NPs aggregates; ordered assemblies of NPs as superstructures, supercrystals, clusters, and defined superstructures in 2D and 3D are commonly used in SERS as sensing probes [71].

In this way, great interest has arisen from the development of sensing methods for AD biomarkers, based on SERS nanoprobes as an alternative to fluorescent dyes, which use different strategies in their construction to improve sensitivity and selectivity in the quantitative detection in human fluids, such as serum or CSF [72].

#### 4.1.1. SERS Biosensors for Aβ Detection

El-Said et al. developed a SERS antibody-antigen assay to determine the concentration of the Aβ_1–40_ peptide. This Aβ_1–40_ antibody was immobilized on indium tin oxide (ITO) substrate, modified with AuNPs, and showed the highest intensity in SERS spectra due to the interactions between the antigen and antibody at the AuNPs array height of 91 nm, with a good correlation between the intensity of the SERS signals and the Aβ antigen concentration over a range of 100 fg/mL^−1^ μg/mL [73].

**Figure 4:**
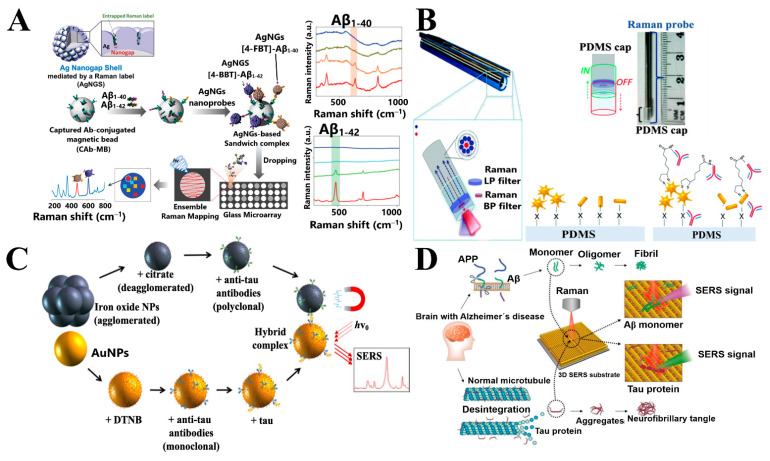
SERS nanoplasmonic platforms for AD biomarkers. (**A**) Schematic illustration for the multiplexed detection of Aβ_1–40_ and Aβ_142_, using Aβ antibody-conjugated AgNGSs nanoprobes in a sandwich immunoassay. Representative Raman spectra for Aβ_1–40_ and Aβ_1–42_ were obtained with the proposed system. Reproduced with permission of Wiley Materials from ref. [72]. (**B**) Graphic description of NPs-modified PDMS caps which can be inserted or removed from the distal end of the Raman probe using AuNSts and AuNRs on PDMS substrates. Republished with permission of Royal Society of Chemistry from ref. [63]. (**C**) Schematic depiction illustrating the formation of the hybrid complex with the individual experimental steps. Reproduced with permission of Wiley Materials from ref. [81] (**D**) Graphical scheme of 3D SERS-substrate for sensitive Aβ and tau detection. Adapted with permission from ref. [86]. Copyright 2020 American Chemical Society.

Another sandwich immunoassay was reported by Yang et al. Silver nanogap shells (AgNGSs) functionalized with Aβ antibody were synthesized as SERS nanoprobes for the sensitive, selective, and multiplexed detection of Aβ_1–40_ and Aβ_1–42_ in the blood (Figure 4A). By finely controlling the formation of AgNGSs as plasmonic “hot-spots” on silica nanoparticles (SiNPs) at the one-nanometer resolution, Aβ_1–40_ and Aβ_1–42_ peptides were detected (Figure 4A) with a LOD of 0.25 pg mL^−1^ and 0.33 pg mL^−1^, respectively, which is one order of magnitude lower than the ELISA test. Moreover, the AgNGSs nanoprobes-based assay has a dynamic range for Aβ detection that is two orders of magnitude higher than ELISA [72].

The detection of small amounts of protein aggregates and the simultaneous knowledge and understanding of conformational changes at low concentrations by a nanofluidics-based SERS detection method was proposed in 2011 by Choi et al. [74]. Based on this study, Choi et al. reported the use of a label-free SERS microfluidics device with 80 nm AuNPs onto a 3-aminopropyltrimethoxysilane (APTMS)-modified glass slide as a SERS active substrate to detect Aβ_1–40_ aggregates and investigate the aggregation process, including the formation, consumption of α-helix intermediates and growth to β-sheet structures at a low concentration range of 10 fM to 1 μM. With these results, the authors indicate that protein aggregation and its resulting conformational characteristics depended on the initial Aβ concentration, accessing to observe the early stages of the Aβ aggregation process and better understand the conditions which lead to NDDs, such as AD [75].

Another study that used SERS-based microfluidic devices was recently published by Dallari et al. This device is composed of a low-cost polymeric replica molding (REM) with 3D printing stereolithography processes, where -citrate (cit) and -lipoic acid (LA) capped gold nanospheres (AuNSps) and gold nanostars (AuNSts) were deposited and modified with polydimethylsiloxane (PDMS), leading to the formation of self-assembled monolayers (SAMs). This AuNPs-PDMS device was coupled with a portable Raman fiber probe and tested in an aqueous solution of Rhodamine 6G (Rh6G) and 4 μM of Aβ_1–42_ peptide. Consistent with Rh6G results, the Aβ Raman spectra showed that the intensity of characteristic peaks was higher in the presence of metallic nanostructures (with which the plasmonic band is in resonance with the excitation wavelength), while for bare PDMS, the Raman profile was almost not visible. A 2 to 5-fold increase in the intensity of some Aβ Raman signals was observed for cit-AuNSts and LA-AuNSts, respectively, due to the presence of “hot-spots” [76].

On the other hand, the concept of SERS-on-fiber technology for diagnostic purposes has been recently developed by the same research group [63]. The integration of a cap SERS-active substrate composed of AuNSps, AuNSts, and CTAB-capped gold nanorods (AuNRs) is assembled in PDMS on the tip of an optical fiber, as shown in Figure 4B. Of the three types of NPs used, AuNSts modified- PDMS caps obtained the highest values of AEF due to the shape of these nanostructures increases the possibility of “hot spots” formation. Functionalization of the NPs with a specific antibody for the Aβ_1–42_ monomer (anti-Aβ) allowed SERS detection of Aβ at the μM level, obtaining an increase of 100-fold in Raman signals in the NPs-decorated caps, with similar behavior for AuNRs and AuNSts, respectively [63]. These results show that metal nanostructures are useful for the detection of target analytes and generate a SERS-enhancement effect.

Compared to the conventional SERS substrates, the use of three-dimensional (3D) nanostructures has been explored to increase the density of metal nanostructures that support LSPR [77]. Nanowire arrays (NWs), acting as frameworks, show a Raman signal sensitivity significantly higher than that of a planar framework of AuNPs two-dimensionally adsorbed on a flat surface [77]. Lin et al. fabricated ordered hexagonal-packed silicon nanorod arrays (SiNRs) functionalized with AuNPs (SiNRs@AuNPs) as SERS substrates for the ultrasensitive detection of Aβ aggregates. By tunning the gap, diameter, and length of the SiNRs, the substrate exhibits stable and reproducible Raman signals, allowing its application in the detection of long Aβ fibrils at a single fibril level, which reveals the secondary structure of Aβ fibrils with high sensitivity and provides a potential method for the study of the dynamic interaction of label-free amyloid protein [77].

Recently, Xia et al. have developed bifunctional NPs for detecting and imaging purposes. AuNPs were functionalized with Rose Bengal dye (RB-AuNPs) and allowed the SERS-based in vitro detection of Aβ_1–42_ between 0–2 μM, showing high selectivity due to the affinity between RB and Aβ [78]. In addition, confocal imaging of amyloid plaques in brain slices from transgenic mice revealed an age-dependent amyloid deposition [78], consistent with previous studies [79].

#### 4.1.2. SERS Biosensors for Tau Detection

Zengin et al. developed a system based on silica-coated magnetic NPs (γ-Fe_2_O_3_@SiO_2_) functionalized with monoclonal anti-tau antibody and sandwiched with AuNPs modified with polyclonal anti-tau and 5,5-dithiobis (2-dinitrobenzoic acid) -DTNB- for the ultrasensitive detection of tau protein. Thus, the SERS intensity peak at 1332 cm^−1^ of DTNB allowed the detection of tau with a LOD lower than 25 fM [80].

In comparison to Zengin et al., Maurer et al. reported a sandwich assay to detect tau in CSF, simplifying the magnetic component by Fe_x_O_y_ NPs functionalized with polyclonal anti-tau antibody (Figure 4C). This approach reduced synthesis time and the workload for preparing the whole tau protein-specific immunosensor and can be transferred to other protein biomarkers, by exchanging the specific antibody [81].

#### 4.1.3. SERS Biosensors for the Simultaneous Detection of Aβ and Tau

Sinha et al. designed magnetic core-plasmonic gold shell NPs decorated hybrid 3D graphene oxide (GO) as a robust 3D SERS platform with a high number of “hot-spots” for the detection of Aβ and tau in whole blood samples. The AEF value for the 3D SERS substrate was about 5 orders of magnitude higher than that of plasmonic NPs (0D) alone and more than 9 orders of magnitude higher than 2D GO, and allowed the high capture and fingerprint identification of trace levels of Aβ and tau protein, with a LOD of 500 fg/mL and 0.15 ng/mL, respectively [82], values which are several orders of magnitude higher than the currently used technology in clinics.

Another highly sensitive and selective SERS-platform was reported by Demeritte et al. Iron-gold core-shell NPs attached to hybrid graphene oxide (HGO) allowed the selective separation and accurate identification of Aβ and tau from whole blood samples. The results revealed that the multifunctional HGO nanoplatform can be used as a SERS “fingerprint” for Aβ and tau detection in concentrations as low as 100 fg/mL, with a LOD several orders of magnitude higher than that of the ELISA kit for Aβ (0.312 ng/mL) and tau protein (0.15 ng/mL) [83]. The high sensitivity obtained is attributed to strong plasmon-coupling, which generates huge amplified electromagnetic fields at the “hot spots”. Furthermore, the nanoplatform can distinguish Aβ and tau biomarkers from human serum albumin (HSA), which is one of the most abundant proteins in the CSF.

Recently, Park et al. created a carboxylic-acid-functionalized and graphitic nanolayer-coated label-free 3D-SERS sensing platform (Figure 4D) composed of well-defined and uniform gold nanowire arrays (AuNWs). An AEF of 5.5 × 10^5^, comparable with reported AEF values for similar 3D SERS substrates [84,85], allowed the detection of conformational changes and the concentration of Aβ and tau proteins, with linear ranges of 10^−7^ M to 10^−9^ M and 10^−8^ M to 10^−11^ M, respectively [86].

Hybrid bionanomaterials, such as DNA-AuNPs conjugates, provide excellent platforms for chemical and biological applications. In particular, the use of poly adenine (polyA) can serve as an anchoring block for preferential binding with AuNPs surfaces [87], and the appended recognition block can form an upright conformation that benefits DNA hybridization. These nanomaterials have been applied for the detection of DNA and small molecules, but not proteins. However, Zhang et al. developed a robust and sensible SERS biosensing platform for the detection of AβOs (Aβ_1–42_) and tau. Using different Raman dye-coded (polyA) aptamer-gold nanoparticles (PAapt-AuNPs) conjugates, the specific protein-aptamer binding induced the aggregation of AuNPs and the plasmonic coupling effect of “turning on” SERS detection of target biomarkers. This strategy displayed excellent analytical performance for the detection of AβOs and tau, with a LOD of 3.7 × 10^−2^ nM and 4.2 × 10^−4^ pM, respectively. In addition, AβOs and tau were detected in artificial CSF (aCSF), with high recovery percentages (96−98% and 97−99%, respectively) [88].

#### 4.1.4. SERS Biosensors for Other Biomarkers

A recent clinical study in AD patients was reported by Carlomagno et al., who developed an innovative SERS-based biosensor using 35 nm AgNPs to analyze human serum from AD patients well-characterized from an A/T/N (A = amyloid, T = tau; N = neurodegeneration) perspective, versus healthy subjects. After optimizing different parameters, the SERS results demonstrated a statistical difference between the spectra collected from the two experimental groups, with accuracy, precision, and specificity of 83%, 86%, and 86%, respectively. On the other hand, the authors found a direct relationship between brain tissue degeneration visualized by magnetic resonance imaging (MRI) and the appearance of a specific biochemical pattern from SERS data in serum collected from AD patients, showing that SERS is a potential label-free tool for monitoring AD progression and rehabilitation treatments in a minimally invasive way [89].

### 4.2. Fluorescence-Based Sensing Platforms

Fluorescence-based biosensors have also been developed for the detection of different biologic analytes. Using plasmonic NPs, the LSPR can generate two effects on the emission of a fluorophore, depending on the fluorophore-nanoparticle distance: fluorescence quenching or enhanced fluorescence intensity [52]. Thus, if the distance is less than 5 nm, fluorescence quenching occurs, due to the energy of the excited fluorophore is totally transferred to NP [52]. For distances from 5 nm or more, an increase in the fluorescence of a quantum emitter has been reported. This enhancement effect in fluorescence due to the presence of metal NPs has been attributed mainly to two factors: (i) increase in the excitation efficiency of the molecules placed near to local electric fields of the NPs and (ii) increase in the radiative decay rate of the fluorophore due to re-radiation by the plasmonic NPs [52].

#### 4.2.1. Fluorescent Biosensors for Aβ Detection

**Figure 5:**
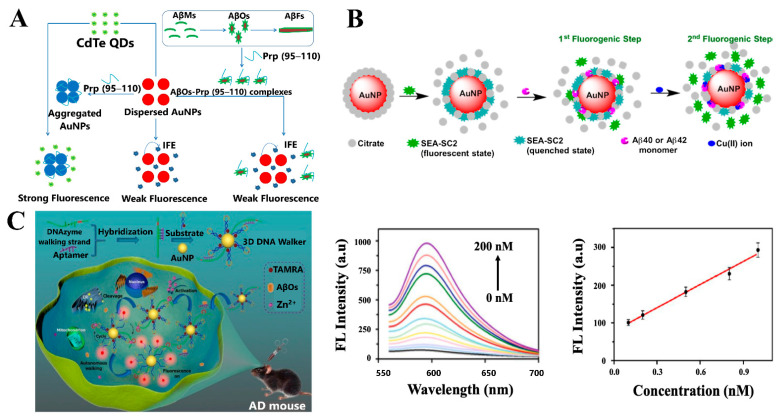
Examples of fluorescent sensors using NPs. (**A**) Schematic illustration of the visual and fluorescent detection of AβOs through the inner filter effect (IFE) of AuNPs on the fluorescence of CdTe-QDs. In the presence of AuNPs, the emission of CdTe-QDs is significantly quenched. The AβOs-PrP (95–110) complex can be adsorbed onto the surface of the AuNPs, thus triggering the aggregation and color change of the AuNPs suspension. As a result, the IFE of AuNPs on the fluorescence of CdTe-QDs is weakened and fluorescence intensity is recovered. Upon addition of Aβ samples to the PrP (95–110) solution, PrP (95–110) interacted specifically with AβOs, thus losing the capability to induce AuNPs aggregation. Reprinted from ref. [90], Copyright 2016, with permission from Elsevier. (**B**) Strategy for the detection of the Aβ monomer in the presence of AuNPs and the small fluorophore SEA-SC2. Reprinted from ref. [91], Copyright 2020, with permission from Elsevier. (**C**) Schematic illustration of AβOs sensitive assay, based on the target-triggered DNAzyme-driven 3D DNA walker fluorescence signal amplification technology; fluorescence spectra of the 3D DNA walker nanoprobe in the presence of different concentrations of AβOs (0~200 nM) and linear plot of fluorescence intensity versus the corresponding AβOs concentrations. Reprinted with permission from ref. [92]. Copyright 2020 American Chemical Society.

Xia et al. reported a label-free fluorescent detection method for AβOs based on the quenching efficiency of AuNPs through the inner filter effect (IFE) on the fluorescence of cadmium telluride quantum dots (CdTe-QDs), as illustrated in Figure 5A. This method enabled the quantification of AβOs with a LOD of 0.2 nM, with high selectivity, due to the specific recognition of AβOs by PrP (95–110) [90].

Saini and Sadhu reported that AuNPs quenched the fluorescence of the SEA-SC2 probe, which was recovered after treatment with Aβ monomers, showing a stronger affinity between Aβ monomers and AuNPs, compared to their affinity towards SEA-SC2. Furthermore, in the presence of Cu^2+^ ions, the formation of the Aβ-Cu^2+^ complex resulted in a greater rapid fluorescence enhancement using SEA-SC2 (Figure 5B). Thus, this method allowed the detection of Aβ_1–40_ and Aβ_1–42_ monomers at pH 7.4 in a concentration range of 10–60 nM. In addition, the methodology was used to evaluate aCSF and HSA samples and revealed that the detection of Aβ_1–40_ monomers was 1.5 times more efficient than for Aβ_1–42_ at 60 nM in aCSF [91].

DNA molecular machines, in particular, DNA walkers, are considered attractive amplification strategies due to the advantage of the signal enhancement cascade generated by its autonomous movement [92]. Yin et al. designed a 3D DNA walker nanoprobe for the sensitive detection and real-time in situ imaging of AβOs. The DNA walker nanoprobe composed of AuNPs modified with aptamer-Zn^2+^-dependent DNAzyme walking strands and TAMRA-labeled hairpin substrate strands (Figure 5C) enabled the in vitro fluorescence detection of AβOs in the concentration range of 0.1–1.0 nM, with a LOD of 22.3 pM, as shown in Figure 5C [92].

Based on the enhancement fluorescence effect, Jara-Guajardo et al. reported that AuNRs functionalized with D1 peptide (AuNRs-PEG-D1), and through the measurement of the fluorescence intensity of the near-infrared (NIR) fluorescent probe CRANAD-2, improved the in vitro detection of Aβ_1–42_ aggregates. AuNRs-PEG-D1 at concentrations of 0.1 nM and 0.5 nM increased the fluorescence of CRANAD-2 bound to Aβ fibrils (2 and 3-fold, respectively), allowing its detection at μM concentrations. Furthermore, the methodology was validated ex-vivo in brain slices of transgenic mice and allowed the detection of amyloid plaques, which accounts for the potential applicability of this detection technique to living organisms [93].

#### 4.2.2. Fluorescent Biosensors for Other AD Biomarkers

Han et al. developed a chemical tongue sensor array using 4 kinds of fluorescent gold nanoclusters (AuNCs), with different surface properties for protein discrimination in the nM range. A linear discrimination analysis (LDA) based on fluorescence intensity response patterns was used and 8 proteins with diverse isoelectric points and molecular weights were effectively discriminated in the urine. In addition, the specific protein types detected in serum samples from AD patients could be discriminated from those of osteoarthritis patients and healthy people [94].

### 4.3. Colorimetric Detection

Colorimetric sensors are widely used for the detection and quantification of different analytes. Because the colorimetric response can be followed by visualizing the color change of a solution with the naked eye, this technique is very attractive due to its simplicity (without the use of sophisticated instruments), speed, and low-cost compared with other detection methods [95]. Particularly, the use of plasmonic NPs, such as AuNPs and AgNPs, is widespread in colorimetric assays due to the characteristic color change following mode shift (aggregation and non-aggregation) in the presence of a target analyte, which would allow its application for the detection of different AD biomarkers.

#### 4.3.1. Colorimetric Biosensors for Aβ Detection

**Figure 6:**
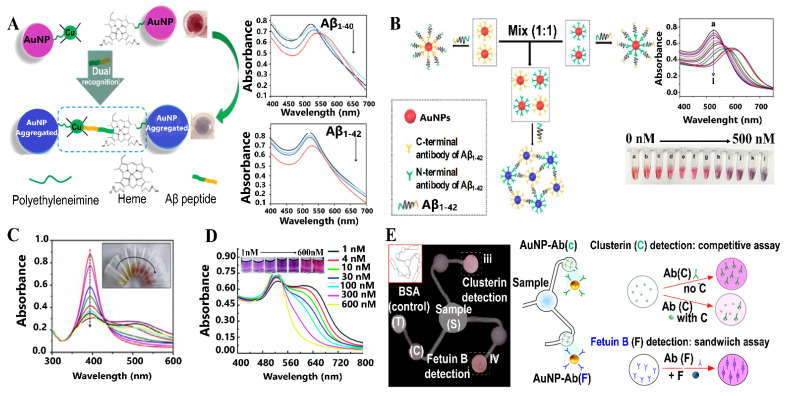
Different colorimetric sensing platforms using NPs. (**A**) Colorimetric sensing of Aβ based on the PEI/AuNPs-Cu-hemin probe; UV-Vis spectra of the PEI/AuNPs-Cu-hemin probe before (in black) and after incubation with different concentrations of Aβ_1–40_ and Aβ_1–42_. Republished with permission of Royal Society of Chemistry, from ref. [96]. (**B**) The principle of the AuNPs@C/N-Ab_1–42_ colorimetric sandwich immunosensor for Aβ_1–42_; absorption profile of the AuNPs@C/N-Ab_1–42_ colorimetric sandwich immunosensor system with increasing concentrations of Aβ_1–42_, from 0 to 500 nM. Reprinted from ref. [99], Copyright 2017, with permission from Elsevier. (**C**) Absorption profile of the Cu^2+−^Ab-AgNPs colorimetric immunosensor with increasing concentrations of Aβ_1–40_/_1–42_, from 0 to 300 nM. Reprinted from ref. [100], Copyright 2016, with permission from Elsevier. (**D**) Absorption spectra of the AuNPs-based colorimetric assay in the presence of various concentrations of AβOs. Republished with permission of Royal Society of Chemistry, from ref. [101]. (**E**) Lateral flow paper device for the detection of clusterin and fetuin B, including sample (S), conjugation (C), and test (T) areas. The selective antibodies for clusterin (C) and fetuin B (F) are immobilized on AuNPs and loaded on the conjugation zone. The strategy used to detect clusterin was based on a competitive assay in which clusterin immobilized on the test zone competes for the AuNPs-Ab with the protein present in the sample. For fetuin B, a sandwich strategy is used, relying on antibody immobilization on the test zone and a combination of analyte and AuNPs-Ab on this zone. Reprinted with permission from ref. [33]. Copyright 2019 American Chemical Society.

Ferriprotoporphyrin IX (Heme) has been associated with AD because it can bind through 3 histidine residues to Aβ forming a heme-Aβ complex, which causes a stabilization of the Aβ peptide structure and therefore inhibits its aggregation process. Moreover, Cu^2+^ can be linked to Aβ through 2 histidine residues, thus forming a Cu-Aβ complex. Yu et al. developed a rapid and sensitive colorimetric method to determine total monomeric Aβ in a rat model of AD. Polyethyleneimine gold nanoparticles (PEI-AuNPs) modified with Cu^2+^ and hemin (PEI/AuNPs-Cu-hemin) allowed the detection of Aβ species through double molecular recognitions, forming a Cu-Aβ-hemin complex and resulting in the aggregation of the NPs with a color change from red to blue (Figure 6A). This method displayed high sensitivity towards total Aβ monomers (Aβ_1–16_, Aβ_1–40_ and Aβ_1–42_) (Figure 6A) within a linear range from 0.2–5000 ng mL^−1^ and presented a LOD ~ 40 pg mL^−1^ [96]. Zhou et al. reported a simpler colorimetric sensing system for Aβ_1–40_ detection in aqueous media based on the aggregation of AuNPs in the presence of Cu^2+^ [97]. The AuNPs conjugated with Aβ_1–40_ and coordinated with Cu^2+^ generated a color change from red to blue, according to the aggregation of the AuNPs, and allowed the detection of Aβ_1–40_ with a LOD of 0.6 nM and application in human serum with acceptable accuracy.

Ghasemi et al. established a label-free colorimetric detection of Aβ_1–40_ and Aβ_1–42_. Conjugated AuNPs and AgNPs with Aβ_1–40_ and Aβ_1–42_ peptides and coordinated with Cu^2+^, produced the aggregation of NPs and therefore different color changes. With this method, was possible the detection and discrimination of both Aβ_1–40_ and Aβ_1–42_ from human serum albumin (HSA) between 50 nmol L^−1^–500 nmol L^−1^ concentration levels. Also, this colorimetric sensor allowed the detection of similar Aβ peptides in plasma samples [98].

On the other hand, Hu et al. developed a colorimetric sandwich immunosensor based on AuNPs coated with -N and -C terminal Aβ_1–42_ antibodies (AuNPs@C/N-Ab_1–42_), which can detect Aβ_1–42_ owing to the specific binding of N- and C-terminal antibodies, generating the aggregation of AuNPs@C/N-Ab_1–42_, which produce a color change from red to blue (Figure 6B). With a linear range from 7.5–350 nM and LOD of 2.3 nM, which is equal or better than other reported methods, this immunosensor allows simple and specific detection of Aβ_1–42._ Furthermore, it can be applied in real blood samples, with high recovery percentages [99]. The same research group also developed a similar immunosensor with AgNPs. This study used a C-terminal Aβ_1–40/1–42_ antibody conjugated with AgNPs (Ab-AgNPs) for the specific and selective detection of Aβ_1–40/1–42_. The binding between Aβ_1–40/1–42_ peptides and Cu^2+^ produces the aggregation of Ab-AgNPs, and a color change from yellow to red (Figure 6C). This immune-sensing approach detected Aβ_1–40/1–42_ in the 0.25–150 nM concentration range, with a LOD of 86 pM, which is equal or better than LODs reported for other assays (ELISA and others). Finally, the immunosensor was applied to real blood samples, with high recovery percentages (~99%) [100].

On the other hand, Zhu et al. developed a colorimetric aptasensor based on cit-AuNPs for the detection of AβOs (Aβ_1–40_), by monitoring the UV-Vis spectrum in the aggregation/stabilization of the AuNPs-aptamer in the presence of AβOs (Figure 6D). This aptasensor presented a linear range of 1–600 nM and a LOD of 0.56 nM, and detected AβOs in aCSF samples, with recovery percentages between 92–101% [101].

Li et al. used polyoxometalate (POM)/Aβ_15–20_ coated-AuNRs for the colorimetric detection of Aβ_1–40_ aggregates. The Aβ_1–40_ aggregation process can be visualized by the naked eye by observing the color change from brown to blue produced when the Aβ aggregates induce AuNRs precipitation; this produces a shift in the LSPR that intensifies as the concentration of Aβ_1–40_ aggregates increases [102]. Moreover, the proposed system was applied as a chemotherapeutic agent and for the photothermal treatment of AD.

#### 4.3.2. Colorimetric Biosensors for the Detection of Other AD Biomarkers

Colorimetric methods have also been proposed for the detection of other AD biomarkers.

miR-137 has demonstrated its potential as a non-invasive biomarker in blood for AD diagnosis and prognosis [36]. Delkhahi et al. reported a new sensitive and specific microRNA assay based on colorimetric detection of AuNPs using DNA hybridization chain reaction amplification (HCR). This method can detect miR-137 with a LOD of 0.25 nM [36], allowing its application in real blood samples without the need for sample preparation, RNA extraction, and/or amplification.

Altered concentrations of proteins such as Fetuins and Clusterin have been related to AD [33]. Considering these antecedents, Brazaca et al. developed a microfluidic paper-based analytical device (μPAD) with colorimetric detection, using selective antibodies immobilized on AuNPs for the simultaneous quantification of Fetuin B and Clusterin (Figure 6E). Through the analysis of color intensity, Fetuin B and Clusterin were detected in the range of 0.1–500 nM and 0.1–1000 nM, with a LOD of 0.24 and 0.12 nM, respectively [33], making this biosensor a great potential for the early detection of AD.

Ren et al. reported a sandwich-type colorimetric immunosensor for the detection of ApoE, using specific anti-ApoE “nanobodies” as the capture and detection antibodies on ITO-AuNPs surface and a gold-TiO_2_-glucose oxidase (GOx) support. Through the catalysis of glucose to H_2_O_2_ by GOx, ApOE was detected with a LOD of 0.42 pg/mL, and the clinical analysis achieved a satisfactory performance [103].

### 4.4. Localized Surface Plasmon Resonance Detection Platforms

LSPR is a particular type of SPR technique. The localized plasmons in metal NPs are formed by coupling with photons at certain wavelengths. Thus, the signal transduction mechanism of LSPR platforms is based on their sensitivity to local refractive index changes (as a shift of extinction wavelength) near the surface of -size and -shape-controlled AuNPs and AgNPs after specific recognition between a biomarker and a recognition element [40,104]. So far, some studies have reported the use of LSPR for the detection of AD in fluids.

#### 4.4.1. LSPR Biosensors for Aβ Detection

**Figure 7:**
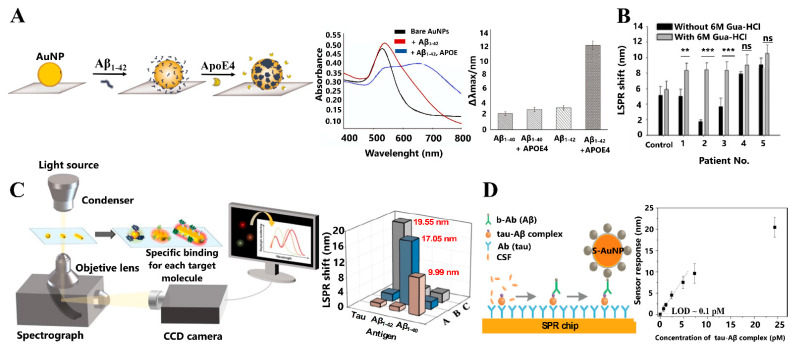
Different sensors for LSPR detection of AD biomarkers. (**A**) APOE4-mediated β-amyloid aggregation sensor. UV–vis absorption spectra of AuNPs after exposure to Aβ and ApoE4 with their respective LSPR λ_max_ shifts (nm). Reprinted from ref. [105], Copyright 2015, with permission from Elsevier. (**B**) LSPR shifts (nm) of the nanoplasmonic sensor to detect tau in blood samples from controls and AD patients (patients no. 1−5), combined with 6 M Gua-HCl. Data are means ± SD from three independent experiments (*n* = 20 single AuNRs examined for each experiment). ** *p* < 0.001, *** *p* < 0.0001 (Student’s *t*-test). Reprinted with permission from ref. [106]. Copyright 2019 American Chemical Society. (**C**) Schematic illustration of a shape-code plasmonic biosensor with their LSPR peak shift (nm) for the independent detection of Aβ_1–40_, Aβ_1–42_, and tau. Reprinted from ref. [107], Copyright 2018, with permission from Elsevier. (**D**) Scheme of the assay for the detection of the tau-Aβ complex and calibration curve for the S-GNP-enhanced detection of the tau-Aβ complex in 10% CSF. The sensor response is expressed in terms of a shift (nm) in the wavelength at which the SPR dip occurs and is proportional to a change in the refractive index caused by the binding of molecules to the sensor’s surface. Reprinted from ref. [108], Copyright 2020, with permission from Elsevier.

Kang et al. developed an AuNPs-LSPR sensor for Aβ_1–42_ by inducing its aggregation with ApoE4. The AuNPs acted as a catalytic activator and optical reporter, while ApoE4 acted as a chaperon to monitor Aβ_1–42_ aggregation, with significant changes in the UV-Vis spectra in the presence of ApoE4 (blue curve, Figure 7A. Under physiological conditions (CSF), the LOD was 1.5 pM [105], demonstrating that ApoE4 induces Aβ_1–42_ aggregation with high specificity, compared to Aβ_1–40_ (Figure 7A).

#### 4.4.2. LSPR Biosensors for Tau Detection

Recently, Kim et al. proposed an aspect-ratio-3.67 AuNRs-mAb as an LSPR biosensor to detect tau in human plasma. To enhance detection sensitivity, guanidine hydrochloride (Gua-HCl) was used as a chaotropic agent, which interrupts water molecules networks and weakens the hydrophobic interactions between proteins. This allowed the ultrasensitive detection of tau in plasma in the fM range, with a LOD of approximately 100 fM [106]. In addition, this system can accurately diagnose AD in blood samples from AD patients due to the higher LSPR shifts produced with the chaotropic agent, compared to the shifts produced in its absence (gray and black bars in Figure 7B, respectively).

#### 4.4.3. LSPR Biosensors for the Simultaneous Detection of Aβ and Tau

A multiplexed LSPR immunosensor for Aβ_1–40_, Aβ_1–42_, and tau using 50 nm AuNSps, and AuNRs with aspect ratios of 1.6 and 3.6, modified with their respective antibodies was developed by Kim, et al. (Figure 7C). Depending on the shape of the NPs (sphere or rod) and the aspect ratio of the AuNRs, the determination of each biomarker was achieved under physiological conditions of mimicked plasma. Thus, each NP interacted with their corresponding biomarker and generated a significant LSPR peak shift (Figure 7C), allowing detection of Aβ_1–40_ with AuNSps, Aβ_1–42_ with AuNRs of aspect ratio 1.6, and tau with AuNRs of aspect ratio 3.6, with LODs of 34.9 fM, 26 fM, and 23.6 fM, respectively [107].

Recent evidence has indicated that the levels of the complex formed between tau and Aβ (tau-Aβ) could be related to AD progression [108]. Springer et al. developed a new LSPR biosensor for the tau-Aβ complex, based on a sandwich assay that incorporates thiol functionalized AuNPs (S-AuNPs) (Figure 7D). The interaction or binding of the target analyte with the sensor surface, resulted in a change of the refractive index and therefore a shift in wavelength, which allowed the detection of the tau-Aβ complex in 10% of CSF and with a LOD of ~0.1 pM (Figure 7D). On the other hand, a LOD of ~1 pM was obtained from real CSF samples from healthy patients. Although this value is low, it would not allow the detection of the tau-Aβ complex in people with AD whose levels of this biomarker are lower [108]. These preliminary results are a beginning to establish new methodologies to improve the LOD of this AD biomarker.

#### 4.4.4. LSPR Biosensors for Kinetic Studies of Aβ Aggregation

Changes in LSPR can also be applied to kinetic studies of biomolecular interactions. NDDs such as AD exhibit a common characteristic mechanism that involves an aggregation process due to protein misfolding [109,110]. This aggregation process promotes the generation of different kinds of species, starting from monomers that assemble into dimers, tetramers, oligomers, and finally, fibrils [111,112]; the latter corresponds to the stable form of the aggregation process and possesses a highly organized structure of cross-β sheets. The intermediate species have been described as the most toxic ones, which provoke neuronal death [113,114,115]. In fact, the generation of these different aggregation species dictates the onset and progression of the disease.

Commonly, the kinetics of protein aggregation is often inferred from a thioflavin T (ThT) or a Congo red fluorescence assay, which report the following three stages of the pathway that leads to the amyloid state: protein nucleation, elongation, and saturation [116,117,118]. However, these fibril-specific dyes have sensitivities in the μM range [119,120] and are not sensitive to the early formed oligomers, limiting their use in detecting oligomeric species [121].

Due to the high sensitivity of LSPR of metal NPs towards changes in the local refractive index and dielectric properties [121], the use of LSPR as a detection tool for AD would allow the sensitive measurement of NPs-protein interactions and detection of the species formed in the aggregation process in real-time and in a label-free mode.

**Figure 8:**
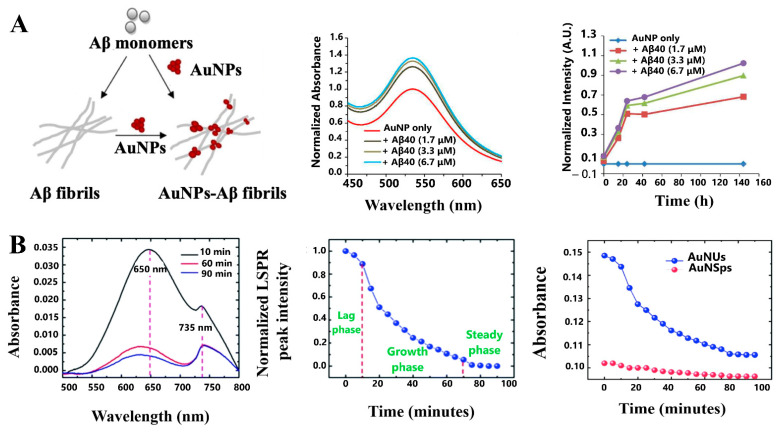
(**A**) Aβ aggregation kinetics studies using AuNPs; UV-Vis spectra of AuNPs measured after 24 h of incubation and Aβ_1–40_ aggregation kinetics followed by the LSPR band intensity change at 535 nm. Adapted with permission from ref. [121]. Copyright 2017 American Chemical Society. (**B**) Absorbance data of samples at 10, 60, and 90 min incubation times. The 650 nm and 735 nm absorbance peaks correspond to AuNUs and Aβ, respectively. LSPR sensing of various fibrillation stages of Aβ_1–42_ and comparison between LSPR sensing of Aβ fibrillation at nM range, using AuNUs (blue) and AuNSps (red). The three figures were obtained from ref. [122] published by The Royal Society of Chemistry.

Elbassal et al. performed a study with sub-micromolar sensitivity to monitor the Aβ_1–40_ aggregation kinetics of AβFs and AβOs using the changes produced in the LSPR band of AuNSps. For AβFs formation, after 24 h incubation of monomers, the intensity of the LSPR band of AuNPs gradually increased, compared with AuNPs-Aβ_1–40_ (Aβ concentration-dependent) and increased with time, according to the fibrillization kinetics of Aβ_1–40_ (Figure 8A). Furthermore, the LSPR band intensity was sensitive to the presence of AβOs for both Aβ_1–40_ and the Aβ_1–40_ mutant Aβ40-K16Nle which forms more stable aggregation intermediates [121].

On the other hand, the progress in the fibrillation process of Aβ_1–42_ at nM concentrations was sensed according to the reduction of LSPR peak intensity of gold nano-urchins (AuNUs). With this method, the LSPR of AuNUs at 650 nm gradually decreased with time (Figure 8B) an effect that was also evident at various fibrillation stages of Aβ_1–42_ (Figure 8B) [122]. In addition, Aβ fibrillation at nM concentrations using AuNSps and AuNUs revealed that AuNSps did not show significant changes in optical absorption compared to AuNUs at various fibrillation stages (Figure 8B), thus limiting its capacity for sensing low concentrations in the fibrillation process.

Table 1 presents an overview of optical biosensors developed in the last decade for the detection of AD biomarkers.

## 5. In Vivo Imaging Detection Platforms of AD Using Plasmonic Nanoparticles

The definitive diagnosis of AD is only possible postmortem through neuropathological demonstration of Aβ plaques and NFTs. For this reason, the promise of “early diagnosis of AD” is the current focus in the development of diagnostic methods for imaging and molecular detection of biomarkers [123].

For brain imaging, the diagnostic modalities include positron emission tomography (PET), x-ray computed tomography (CT), and magnetic resonance imaging (MRI) [124]. Usually, after the appearance of the first clinical symptoms, the use of neuropsychological testing, in conjunction with these imaging techniques, allows one to estimate disease progression. Of the techniques mentioned above, PET and MRI have been the primary imaging modalities used in AD, which allow the assessment of cortical atrophy and amyloid load, respectively. Additionally, glucose metabolism has been measured using fluorodeoxyglucose-PET [125].

In PET, the compounds that have been used as probes for AD detection are the radiotracers (11)C-labeled Pittsburgh Compound-B (11)C-PiB, [18F] Flutemetamol, [18F] Florbetapir [126] that have been approved by the Food and Drug Administration (FDA). Despite this, they have not shown conclusive results as a precise diagnostic methodology and are rather useful for monitoring the disease [126]. These radiotracers do not allow the detection of soluble Aβ species, so it would not be possible to make an early diagnosis, since they only detect insoluble Aβ species [126]. In addition, this technique has a great monetary cost, being necessary to have a nearby synchrotron to obtain the radiotracer. On the other hand, MRI provides excellent soft-tissue contrast and is considered safe because there is no need for ionizing radiation or radioactive traces at any point of the scanning process [124].

Due to the modifiable characteristics of NPs mentioned in Section 3 such as their size, shape, chemical composition, biocompatible coating, and the ability to be functionalized with a targeting ligand, fluorescent probe, or drug, render them as optimal candidates as contrast agents for imaging diagnostic applications in the central nervous system (CNS). For successful identification and imaging of plaques in AD, NPs-contrast agents must be able to cross the blood–brain barrier (BBB) [127]. Furthermore, these agents should maintain colloidal stability, safely interact with organs and display effective renal or hepatic clearance to prevent accumulation and toxic effects [125].

In literature has been reported that the NPs mostly used as contrast agents to improve sensitivity and enable the visualization of Aβ plaques by MRI are those that contain superparamagnetic (SPIONs) and magnetic iron cores (IONPs), which influence spin-spin (T2, transversal) relaxation times of the protons present in water molecules in surrounding tissues. Chelates of Gadolinium (Gd), Gd^3+^ is usually available as standard T1 (longitudinal) MRI contrast agents. In addition, the use of Gd-based small molecules [128,129] and Gd ions in NPs have been used in imaging applications [130,131]. More details about these contrast agents can be found in the interesting reviews by Ulanova et al. [125] and Sharma et al. [124].

In particular, studies regarding the use of plasmonic NPs for AD imaging are very limited. Some ex-vivo studies have been performed using AuNPs, with potential future applications in AD sensing [78,93,132]. However, a major disadvantage of these experiments is that they are not fully representative in antemortem conditions.

**Figure 9:**
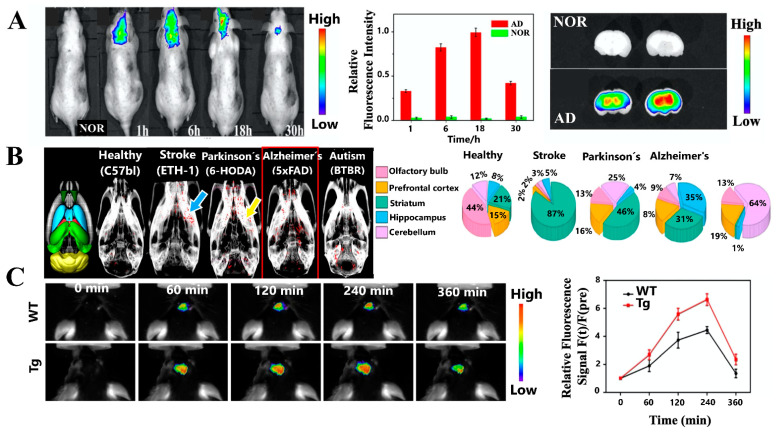
(**A**) Fluorescence imaging upon excitation at 420 nm of the blank control group (NOR) of mice and AD model mice via tail-vein injection of 10 mmol/L HAuCl_4_ solution at 1 h, 6 h, 18 h, and 30 h. The variations of the relative fluorescence intensity in normal control mice and AD model mice indicate that at 18 h the maximum fluorescence intensity occurs. In a transverse section of NOR and AD mice’s brain treated with 10 mmol/L HAuCl_4_ solution at 30 h post-injection; no fluorescence signal was detected in the hippocampus of the NOR mice. Republished with permission of Royal Society of Chemistry, from ref. [133]. (**B**) In vivo CT imaging of AuNPs-loaded MSC-exo demonstrates different homing patterns in healthy controls, stroke, Parkinson’s, Alzheimer’s, and autism models, 24 h post intranasal administration. The light blue arrow indicates the location of the ETH-1 injection and the yellow arrow, the location of the 6-OHDA injection. Relevant brain sections were adapted from the Allen Mouse Brain 3D Atlas (dark green: olfactory bulb; blue: striatum; red: thalamus; green: hippocampus; and yellow: cerebellum). The percentage of the CT signal 96 h post-administration is shown for healthy control mice and stroke, Parkinson’s disease, Alzheimer’s disease, and autism models. Values are normalized to show percentiles, according to the total number of gold voxels in each brain. In AD model, the highest percentages of CT signal are found in the hippocampus and striatum regions. Reprinted with permission from ref. [135]. Copyright 2019 American Chemical Society. (**C**) In vivo fluorescence images and relative fluorescence signal of the WT (top row) and Tg (bottom row) mice at selected time points before and after injection of 3D DNA walker nanoprobe. Reprinted with permission from ref. [92]. Copyright 2020 American Chemical Society.

For in vivo imaging, in a literature search performed on PubMed databases using the following terms: Alzheimer´s disease AND plasmonic nanoparticles AND in vivo imaging, only a few recent studies were found. Due to the fluorescence of AuNCs within the biological window, in 2016 Lai et al. developed a fluorescent method for in vivo imaging of the brain in an AD mouse model. Through the biosynthesis of AuNCs in brain affected sites with AD using an intravenous injection of a gold salt such as chloroauric acid (HAuCl_4_), this method allowed the fluorescence labeling around these sites within a couple of hours, with a maximum at 18 h, compared to normal mouse brains (Figure 9A)**,** which did not exhibit fluorescence. These results suggested that HAuCl_4_ could pass the BBB and reach the brain of the AD mice, where it was observed that the accumulation of AuNCs was mainly in the hippocampus region as shown in Figure 9A [133].

Due to the high attenuation coefficient value of gold (5.16 cm^2^ g^−1^ at 100 keV), compared with contrast agents based on iodine (1.94 cm^2^ g^−1^ at 100 keV) [134], this element could be applied in imaging techniques such as CT. Perets et al. reported the use of intranasally administered exosomes from bone marrow mesenchymal stem cells (MSC-exo) labeled with AuNPs (MSC-exo-AuNPs). Using CT, this methodology allows the in vivo study of AD and other neurodegenerative, neurovascular, and neuropsychiatric diseases, such as Parkinson’s disease (PD), ischemic stroke, and autism. In murine models, this technique allowed track of migration and homing patterns of the MSC-exo, revealing a selective accumulation in areas of the brain correlated with inflammatory mechanisms of these diseases (Figure 9B). Therefore, this system is an alternative to study the advancement of these types of diseases and anticipate more complex stages [135].

More recently, Yin et al. explored the potential of their 3D DNA walker nanoprobe (described in Section 4.2) for fluorescence imaging of AβOs in the APP/PS1 double transgenic (Tg) AD mouse model, compared with C57BL6 wild-type (WT) mice as controls. At all-time points from 30 to 360 min after injecting 2 nM of the nanoprobe, the fluorescence signal in the brain was higher in Tg mice, compared to WT mice (Figure 9C), especially up to 240 min, and then rapidly decayed [92]. So, this nanosystem has great potential as a tool for AD diagnosis.

## 6. Conclusions and Perspectives

Due to the diagnosis of AD is made when irreversible brain damage has occurred, the development of cost-effective, non-invasive, portable, and rapid methods for the detection of AD biomarkers in the early stages and their discrimination from other types of dementia, play a key role for the timely treatment of this disease. In this aspect, the development of biosensors as powerful analytical devices seems necessary and unavoidable. Biosensors as analytical platforms allow highly sensitive and specific detection of a target analyte in a short period of time.

In this review, we have presented a summary of articles published in the last decade focused on the development of biosensors based on optical techniques incorporating plasmonic nanoparticles for the in vitro detection of different biomarkers of AD, which the most common are the core biomarkers Aβ and tau protein.

Regarding this topic, four optical detection techniques (SERS, SEF, Colorimetry, and LSPR) were included because they mainly use plasmonic nanoparticles in their configuration. As mentioned in Section 3, these nanoparticles are very useful since they present the LSPR phenomena, which provides an increase in the local electromagnetic fields in the vicinity of the nanoparticles’ surface and the optical extinction and scattering cross-sections [136]. This property, dependent on the chemical composition, size, shape, and medium in which the NPs are dispersed, allows increasing the signals in detection by SERS and SEF as well as observing color changes due to interaction of the nanoparticles with some analyte of interest or changes in the electromagnetic spectrum by displacement of the absorption bands of the NPs as in the LSPR-based sensors.

For the development of new sensing platforms, several key parameters must be considered. In this aspect, the World Health Organization (WHO), has defined seven parameters: “(i) Affordability, (ii) Sensitivity, (iii) Specificity, (iv) User-friendliness, (v) Rapid and robust, (vi) Equipment-free, and (vii) Deliverable to those in need for such technologies”, defining the acronym ‘‘ASSURED’’ [137].

Of all sensor examples presented in this work, SERS and LSPR platforms are promising techniques that can reach LODs at femtomolar and picomolar concentration levels of an AD biomarker (Aβ peptide, tau protein). In the case of fluorescence and colorimetry detection, LOD are generally at the nanomolar range.

In general, SERS has great advantages as sensitivity, multiplexing capability, and resistance to photobleaching [138]. As described in Section 4, nominal parameters as enhancement factors and limits of detection are often used to compare the efficiency of a SERs sensor. However, currently, it has been reported that it cannot be considered as the only parameter when making a comparison [65]. In this regard, of the articles that report SERS sensors, the use of nanostructures type shells/core-shell and 3D superstructures are those that report the lowest detection limits (pg/mL and even fg/mL) and with enhancements factors in some cases of the order of 10^5^, however, in many of these works do not mention about other validation parameters, such as robustness, recovery, etc. Although this technique provides excellent sensitivity, the cost of the equipment, the need for qualified personnel to interpret results, are disadvantages that must be considered for a future clinical application. Currently, SERS-on-fiber technology can be a good alternative for obtaining portable platforms.

In the case of fluorescence-based sensing platforms using plasmonic nanoparticles, few articles have been reported in the detection of AD biomarkers, probably because most of these assays reach a sensitivity that is not clinically relevant [40]. Furthermore, it is necessary to design probes that allow an affinity for these biomarkers. One possibility could be the use of nanoparticles that present intrinsic fluorescence such as quantum dots (QDs) [139] which have some advantages compared to common fluorescent organic molecules, as tunable fluorescent wavelengths from the visible to red, high photostability [140], could be used in the development of biosensors and even in the field of imaging.

On the other hand, colorimetric techniques have the advantage of being low cost, simple and by the naked eye allows quantitative or semi-quantitative detection of a target analyte through a color change, but its main disadvantage is that it is generally less sensitive [141]. In the case of colorimetric platforms for AD biomarkers, many have limits of detection at the nanomolar level (nM) of concentration, which is not sufficient for clinical applications. It is interesting to note that unlike the other detection techniques presented in this review, colorimetric methods have been reported for sensing other less common AD biomarkers, such as miRNAs or Fetuin and Clusterin.

As the refractive index of metal nanoparticles depends on the size and shape, LSPR based-sensors can be useful since they have some advantages such as multiplexed detection, the possibility of miniaturization which reduces the sample volume to be used, and compatibility with microfluidics and in flow assays [44]. As a result, the LSPR detection of AD biomarkers using plasmonic nanoparticles has allowed an ultrasensitive detection obtaining low detection limits of the order between pM and fM in some cases and has achieved the simultaneous detection of AD biomarkers such as Aβ and tau, so in the future, it is a technique that offers promising characteristics.

With respect to the type of matrix to be analyzed for the detection of AD biomarkers, to date, are commonly in CSF samples, since the fluid contains, in part, brain-derived proteins [25]. However, this is an invasive procedure since CSF analysis requires a lumbar puncture. To overcome these limitations, other biological fluids, such as blood, have been explored for the presence of detectable quantities of the Aβ peptide [25]. However, the use of blood samples remains a challenge because the difference in the level of blood-based Aβ between patients and normal individuals is very small (several 10 pg/mL) [142]. Although various methodologies have been developed to detect AD biomarkers in blood as Aβ, most of them have not achieved validate the clinical utility and have failed to replicate results [25,143]. In addition, for accurate detection of AD biomarkers in clinically relevant fluids generally requires recognition elements such as antibodies, aptamers, among others, which have high binding affinity and selectivity. Recently, the so-called nanobodies have emerged, an antigen-binding fragment with a small size, however, only some specific nanobodies for AD biomarkers detection have been reported [103,144].

It has been reported the development of portable optical sensing platforms [145,146,147]. Miniaturized platforms, as capillary, paper, and fiber-based would be very useful for AD detection due to their low cost, portability, and reduction of sample volume to be analyzed [141], which is important for a potential application in clinical diagnosis and multiplexed detection.

Although optical biosensors developed are still only appropriate for research purposes, transforming these platforms into robust practical tools continues to be another challenge. Apart from the advantages already described, the nanoparticles incorporated in these biosensors can be synthesized ensuring monodispersity in size and shape, as well as being stable against degradation and leaching. The possibility of reusing the sensor is another factor that must be considered for practical applications.

Another field that needs to be exploited is the study of the Aβ aggregation process. Currently, studies that use plasmonic NPs in this field are lacking, even though they can detect Aβ aggregates. Moreover, this type of NPs can determine which stage of the aggregation process is disrupted or sense. This information is valuable because several toxic species are formed in the protein aggregation process. Thus, the detection of these species would allow a rational design and development of a biosensor.

Unquestionably, AD diagnosis is possible only during *a* post-mortem examination of Aβ deposits and NFTs in the brain. Therefore, different diagnostic imaging techniques have been developed and used for observing neuroanatomical changes in AD. One of the most popular techniques to detect local brain functional changes is Positron Emission Tomography (PET). Magnetic Resonance Imaging (MRI) and X-ray computed tomography (CT), on the other hand, allow the assessment of structural changes in brain tissue. Even though they provide satisfying results, these methods are not completely specific and accurate. In this regard, the greatest challenge lies in the development of new tracers and contrast agents able to cross the blood-brain barrier (BBB) and reach the brain. This difficulty has been managed over the years by the use of nanotechnology, as mentioned in Section 5. In this section, we highlighted studies that used AuNPs to obtain a system that overcame the difficulty of reaching the brain, accumulated in areas of interest, and could be visualized by in vivo imaging techniques. These advances represent a great benefit for the research area as they allow expanding the field of diagnosis not only of AD, but also of other complex diseases with a difficult diagnosis. Furthermore, this knowledge has contributed to the generation of less harmful diagnostic systems than those used conventionally. Thus, the development of new nanosystems will allow to decrease the time of imaging taking, improve the quality of image acquisition, improved the information acquired, and especially allow an early diagnosis, which could offer a better quality of life for people affected by AD.

Therefore, plasmonic-based platforms for AD diagnosis offer promising future features. In addition, multidisciplinary research is necessary to develop sensitive and reliable sensors that can be applied clinically and compete with those that are commercially available.

Finally, we encourage researchers to investigate the use of plasmonic NPs in detection methods for other kinds of AD biomarkers. Although there is a main focus on the Aβ peptide, other biomarkers such as tau protein, ApoE4, or miRNAs could also be used to complement AD diagnosis in the development of novel biosensors.

## Figures and Tables

**Table 1:** Overview of optical plasmonic sensors for in vitro detection of AD biomarkers.

Type of Nanoparticle	Detected Biomarker	Sensing Method	Analytical Parameters	Application	Ref.
Hybrid (MNPs/AuNPs)	tau	Antibody sandwich SERS assay	LR: 25 fM–500 nM LOD: <25 fM	-	[80]
AgNGSs	Aβ_1–40_ Aβ_1–42_	Antibody sandwich SERS assay	LOD: 0.25 pg mL^−1^ 0.33 pg mL^−1^	Human serum	[72]
MNPs@Au-HGO	Aβ_1–42_ tau	Antibody SERS assay	LOD: <100 fg mL^−1^	Blood	[83]
AuNPs	AβOs (Aβ_1–42_) tau	Aptamer SERS detection	LOD: AβOs: 3.7 × 10^−2^ nM Tau: 4.2 × 10^−4^ pM	aCSF	[88]
AuNPs	Aβ_1–42_	SERS detection with a fluorescent probe	L.R: 0–2 μM	-	[78]
AuNPs	Aβ_1–16_, Aβ_1–40_, Aβ_1–42_ monomers	Colorimetric label-free detection	L.R: 0.2–5000 ng mL^−1^ LOD: 40 pg mL^−1^ (as total monomers)	CSF/AD rat brain tissues	[96]
AgNPs	Aβ_1–40/1–42_	Colorimetric immunosensor	LR: 0.25–150 nM LOD: 86 pM	Blood	[100]
AuNPs	AβOs (Aβ_1–40_)	Colorimetric aptasensor	LR: 1–600 nM LOD: 0.56 nM	aCSF	[101]
AuNPs	miRNA-137	DNA based biosensor	LOD: 0.25 nM	Blood	[36]
AuNPs	Clusterin Fetuin B	Colorimetric microfluidic device	LOD: 0.24 nM 0.12 nM	-	[33]
AuNPs	AβOs	Fluorescence detection	LR: 0.1~1.0 nM LOD: 22.3 pM	-	[92]
AuNPs/CdTe QDs	AβOs	Fluorescence detection	LR: 1–60 nM LOD: 0.2–0.5 nM	-	[90]
AuNPs	Aβ_1–42_ Aggregates	Surface-Enhanced Fluorescence	μM level	PBS	[93]
AuNRs	Aβ_1–42_, Aβ_1–40_, tau	Shape-code LSPR sensor	LR: 1 × 10^1^–1 × 10^8^ fM LOD: 26 fM	-	[107]
AuNPs	Aβ-tau complex	Antibody sandwich LSPR assay	LOD: 1 pM	CSF	[108]
AuNPs	Aβ_1–42_ Aggregates	LSPR sensor	LOD: 1.5 pM	aCSF	[105]

Abbreviations: LR: linear range, LOD: limit of detection, CSF: cerebrospinal fluid, aCSF: artificial cerebrospinal fluid, MPs: magnetic nanoparticles, AuNPs: gold nanoparticles, AgNGSs: silver nano-gap shells, HGO: Hybrid graphene oxide, Aβ: Amyloid-β peptide, AβOs: Amyloid-β oligomers, CdTe QDTs: Cadmium Telluride quantum dots, PBS: Phosphate-buffered saline.

## Data Availability

Not Applicable.
